# Infectious conjunctivitis caused by *Pseudomonas aeruginosa* isolated from a bathroom

**DOI:** 10.1186/1756-0500-6-245

**Published:** 2013-07-01

**Authors:** Hiroshi Eguchi, Tatsuro Miyamoto, Tomomi Kuwahara, Sayaka Mitamura, Yoshinori Mitamura

**Affiliations:** 1 Department of Ophthalmology, Institute of Health Biosciences, The University of Tokushima Graduate School, 3-18-15, Kuramoto-cho, Tokushima-shi 770-8503, Japan; 2 Department of Microbiology, Faculty of Medicine, Kagawa University, Kagawa, Japan

**Keywords:** *Pseudomonas aeruginosa*, Conjunctivitis, Bathroom, Pulsed-field Gel Electrophoresis

## Abstract

**Background:**

The elucidation of the routes of transmission of a pathogen is crucial for the prevention of infectious diseases caused by bacteria that are not a resident in human tissue. The purpose of this report is to describe a case of suture-related conjunctivitis caused by *Pseudomonas aeruginosa* for which we identified the transmission route using pulsed-field gel electrophoresis (PFGE).

**Case presentation:**

A 38-year-old man, who had undergone surgery for glaucoma 2 years ago previously, presented with redness, discomfort, and mucopurulent discharge in the right eye. A 9–0 silk suture had been left on the conjunctiva. A strain of *P. aeruginosa* was isolated from a culture obtained from the suture, and the patient was therefore diagnosed with suture-related conjunctivitis caused by *P. aeruginosa*. The conjunctivitis was cured by the application of an antimicrobial ophthalmic solution and removal of the suture. We used PFGE to survey of the indoor and outdoor environments around the patient’s house and office in order to elucidate the route of transmission of the infection. Three strains of *P. aeruginosa* were isolated from the patient’s indoor environment, and the isolate obtained from the patient’s bathroom was identical to that from the suture.

**Conclusion:**

The case highlights the fact that an indoor environmental strain of *P. aeruginosa* can cause ocular infections.

## Background

The bacterium *Pseudomonas aeruginosa* is a Gram-negative rod that is commonly isolated from aquatic and terrestrial environments. It is an established nosocomial pathogen in the field of hospital infection control [1-3]. The routes of transmission are well investigated epidemiologically, because it is responsible for severe infections in immunocompromised patients. However, in clinical ophthalmology, the transmission route of *Pseudomonas aeruginos* is not well investigated, even it is known to be able to cause keratitis, some strains produce biofilms on contact lenses [4,5], and endophthalmitis caused by *P. aeruginosa* contracted from surgical equipment has been reported as a devastating complication after ocular surgery [6-8]. With regard to bacterial conjunctivitis in adults, it is most often caused by pathogens derived from the indigenous bacterial flora of the ocular surface. Therefore, *P. aeruginosa*, which is not a resident bacterium of the ocular surface, is rarely isolated from conjunctivitis cases except those of patients who have some sort of artificial devices in the eye [9-11].

Most bacterial conjunctivitis encountered in the clinical setting can be treated by an empiric choice of commercially available antimicrobial ophthalmic solutions without rigorous identification of the species of the pathogen, and the sporadic cases may go unnoticed unless an outbreak occurs. Most cases of exogenous conjunctivitis, such as that caused by *P. aeruginosa,* could be prevented by determination both of the origin and of the transmission route of the causative pathogen. Such prevention would contribute to the proper usage of antimicrobials in the field of ophthalmology. Moreover, it is important for the patients who have undergone intraocular surgery to understand the distribution and the route of transmission of *P. aeruginosa* in order to prevent devastating complications. Herein, we describe a case of suture-related conjunctivitis caused by *P. aeruginosa* after glaucoma surgery in which we elucidated the route of transmission using pulsed-field gel electrophoresis (PFGE).

## Case presentation

**Figure 1:**
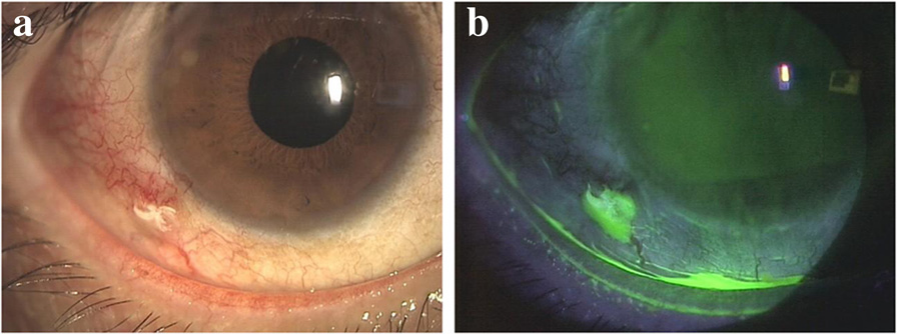
**Conjunctivitis associated with a 9–0 silk. a)** Marked conjunctival hyperaemia and yellowish white discharge around the suture can be seen. **b)** The yellowish white discharge is stained yellow green by fluorescein.

A 38-year-old man, who had undergone a trabeculotomy for secondary glaucoma due to uveitis presented with redness, discomfort, and yellowish white mucopurulent discharge in the right eye. Slit-lamp microscopy revealed conjunctival hyperaemia and mucopurulent yellowish white discharge around a 9–0 silk suture that had been left on the lateral inferior side of the conjunctiva for over 2 years (Figure 1). A Gram-stained smear of the discharge showed polymorphonuclear neutrophils and Gram-negative rods; no eosinophils were observed in a Giemsa-stained smear. The suture was removed from the patient’s eye and cultured, and *P. aeruginosa* was isolated. Drug susceptibility testing using Etest® (bioMérieux SA, Lyon, France) showed that the isolate was susceptible to aminoglycosides (minimum inhibitory concentration (MIC) of tobramycin: 1 μg/ml) and to quinolones (MIC of levofloxacin: 0.25 μg/ml), but resistant to cephems (MIC of ceftriaxone: >256 μg/ml). The conjunctivitis resolved after the application of tobramycin ophthalmic solution 4 times daily for 2 weeks and removal of the suture.

**Figure 2:**
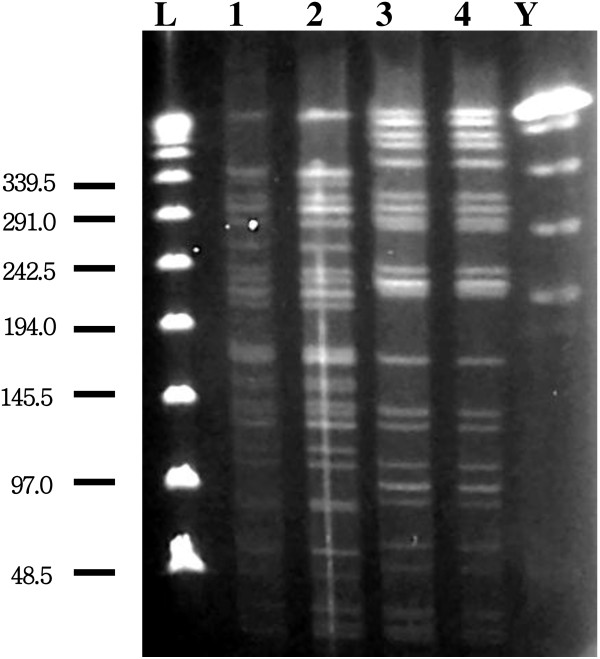
**Pulsed-field gel electrophoresis typing of four *Pseudomonas aeruginosa *strains, one was isolated from the patient’s eye and three, from different environments.** Lane 1 shows the fragment pattern of the *P. aeruginosa* strain obtained from the suture. Lane 2 shows the fragment pattern of the *P. aeruginosa* strain obtained from the patient’s bathroom. Lanes 3 and 4 show the fragment patterns of the strains obtained from the humidifying device and kitchen in the patient’s workplace, respectively. The strain from the suture is identical to that from the patient’s bathroom. ‘L’ represents the lambda ladder; ‘Y’ the yeast chromosome ladder.

To elucidate the route of transmission of *P. aeruginosa* in this case, we first obtained bacterial samples by surveying the patient’s indoor and outdoor environments (with approval from the ethics committee of Tokushima University Hospital). Then, bacterial strains were collected from the environmental samples, and PFGE was performed as previously described [12]. Three strains of *P. aeruginosa* were isolated: from the bathroom in the patient’s home, and the kitchen and a humidifying device at his workplace. The isolate from the 9–0 silk suture was identical that obtained from the patient’s bathroom (Figure 2). Although the other isolates from the workplace were identical to each other, they differed from the isolate obtained from the suture.

### Discussion

Herein we reported for the first time that *P. aeruginosa* isolated from the ocular surface was acquired from the patient’s home environment. This suggests that the bacteria colonising in humid home environments such as the bathroom can infect ocular surfaces in association with biomaterials. Biomaterials such as sutures, punctual plugs, intraocular lenses, and contact lenses can serve as hotbeds for bacterial infections. Therefore, all clinical cases involving the use of biomaterials require regular follow-up. Although we did not examine the biofilm productivity of the strain isolated from this case, many Gram-negative rods, including *P. aeruginosa*, are known to be able to produce biofilms on the surfaces of biomaterials. Knowledge of the distribution and route of transmission of pathogenic microorganisms is important for infection control in patients with biomaterials. Biomaterials around an infectious focus should be removed immediately after detection of the infection if it is deemed necessary and feasible to do so.

*P. aeruginosa* endophthalmitis with disastrous results has been reported following intraocular surgery [7,8], and in these cases the *P. aeruginosa* was determined to have been transmitted from some surgical equipments with which related to water-based supplies. However, the underlying cause of the pollution of the water was not identified. Infections of buckling devices caused by *P. aeruginosa* following retinal detachment surgery have also been described, although the origin and transmission route were not mentioned [9-11]. The shower environment has been described as a source of opportunistic pathogens [13], and we presume that these infections were likely to have been caused by strains of *P. aeruginosa* present in humid indoor areas in the home or hospital to which the patient was exposed. Considering that contaminated rooms may lead to hospital- and community-acquired infections, and also considering that *P. aeruginosa* is strongly associated with environmental contamination [1], disinfection or cleaning of humid home environments may help decrease the risk for infection by *P. aeruginosa*.

## Conclusion

Precautionary protections of the eye from bacterial infections promoted by humid indoor conditions may be important. Removal of ophthalmic biomaterials immediately after their use and application of ophthalmic solutions that target Gram-negative rods derived from indoor humid environment should be considered for cases of conjunctivitis related with some artificial devices on the ocular surface.

## Consent

Written informed consent was obtained from the patient for submission and publication of this case report and any accompanying images. A copy of the written consent is available for review by the Editor of this journal.

## Competing interests

The authors have no conflicts of interest to declare.

## Authors’ contributions

HE diagnosed and treated the patient, isolated the pathogen, conducted the survey of the environment, prepared the plugs for PFGE, performed PFGE and wrote the draft of the manuscript. TM and SM monitor the patient. TK performed the PFGE. YM manages the patient’s entire clinical course. All authors read and approved the final manuscript.
